# Comparing Two Epidemiologic Surveillance Methods to Assess Underestimation of Human Stampedes in India

**DOI:** 10.1371/currents.dis.ab7f298c89854015b74856232c70b62c

**Published:** 2013-09-23

**Authors:** Ka Ming Ngai, Wing Yan Lee, Aditi Madan, Saswata Sanyal, Nobhojit Roy, Frederick M. Burkle, Edbert B. Hsu

**Affiliations:** Department of Emergency Medicine, Icahn School of Medicine at Mount Sinai, New York, New York, USA; Department of Emergency Medicine, New York University School of Medicine, New York, New York, USA; Greater New York Hospital Associations, New York, New York, USA; Jamsetji Tata Centre for Disaster Management, Tata Institute of Social Sciences, Mumbai, India; Jamsetji Tata Centre for Disaster Management, Tata Institute of Social Sciences, Mumbai, India; Jamsetji Tata Centre for Disaster Management, Tata Institute of Social Sciences, Mumbai, India; Harvard Humanitarian Initiative, Harvard School of Public Health, Harvard University, Cambridge, Massachusetts, USA; Office of Critical Event Preparedness and Response, Johns Hopkins University, Baltimore, Maryland, USA

## Abstract

Background: Two separate but complementary epidemiologic surveillance methods for human stampedes have emerged since the publication of the topic in 2009. The objective of this study is to estimate the degree of underreporting in India. Method: The Ngai Search Method was compared to the Roy Search Method for human stampede events occurring in India between 2001 and 2010. Results: A total of 40 stampedes were identified by both search methods. Using the Ngai method, 34 human stampedes were identified. Using a previously defined stampede scale: 2 events were class I, 21 events were class II, 8 events were class III, and 3 events were class IV. The median deaths were 5.5 per event and median injuries were 13.5 per event. Using the Roy method, 27 events were identified, including 9 events that were not identified by the Ngai method. After excluding events based on exclusion criteria, six additional events identified by the Roy’s method had a median of 4 deaths and 30 injuries. In multivariate analysis using the Ngai method, religious (6.52, 95%CI 1.73-24.66, p=0.006) and political (277.09, 95%CI 5.12-15,001.96, p=0.006) events had higher relative number of deaths. Conclusion: Many causes accounting for the global increase in human stampede events can only be elucidated through systematic epidemiological investigation. Focusing on a country with a high recurrence of human stampedes, we compare two independent methods of data abstraction in an effort to improve the existing database and to identify pertinent risk factors. We concluded that our previous publication underestimated stampede events in India by approximately 18% and an international standardized database to systematically record occurrence of human stampedes is needed to facilitate understanding of the epidemiology of human stampedes.

## BACKGROUND

Human stampedes occur globally, affecting both developed as well as developing countries. The number of events in the human stampede database described previously, referred to as the Ngai Search Method, has increased from 215 to 350 between 1980 and 2012.^1^ Globally, a total of 10,243 deaths, 22,445 injuries occurred in 83 countries were recorded in the database as of December 31, 2012. Some high profile events during the past five years include the Cambodian Bon Om Touk Stampede with over 347 deaths,^2^ the Ram Janki Temple stampede with 63 deaths,^3^ and the German love parade with 21 deaths.^4^^,^ 5 However, minimal attention was given to several high mortality events like the Sabarimala stampede which took place in January, 2011 in Kerala, India with 102 deaths,^6^ as well as the Port Said soccer match stampede that occurred in February , 2012 in Egypt with 73 deaths.^7^ The authors believe that human stampedes are more widespread than reflected by this search. Rather, it is the absence of standardized reporting that may account for under recognition.

An international conference in Saudi Arabia in 2010 proposed a research agenda framework for mass gatherings, identifying assessment of risk, including that posed from human stampedes as targeted priorities.^8^ Despite the growing attention focused on crowd disasters, systematic study and prevention of human stampedes remain very limited.^2^^,^ 3 ^,^ 9

During the 17^th^ World Congress on Disaster and Emergency Medicine in Beijing in 2011, two groups of researchers met to compare their search methods for human stampede events in India, a country with the highest incidence of events. The objective of this paper is to estimate the degree of underreporting by comparing the two epidemiologic surveillance methods and to identify additional sources that will strengthen the inclusiveness of the existing global stampede database.

## METHODS

**The Ngai Search Method for Global Human Stampede Events**

The Ngai search method described previously, consists of a primary search performed using a LexisNexis® Academic with the search terms “stampede” & “Injur*”, and Headline (stampede), source: News (all, English, full text) with the option “all available dates.”^1^ Any events involving fire, bombing, or terrorism were excluded. All of the articles were read and reviewed using “full with indexing.”

A secondary hand-search was performed on multiple internet-based English–language news agencies including the British Broadcasting Corporation (BBC) News, the New York Times, Arab News, Cable News Network (CNN), and Reuters. Finally, supplemental internet searches were conducted on PubMed, Wikipedia, crowddynamics.com, and mapreport.com.

The abstracted data, using a previously published format, included: date of event, country, geographical region, time of occurrence (day vs. night), type of event (sports, religious, music/movie, political, non-sports nonreligious nonmusic/movie nonpolitical [non-SRMP]), number of participants, number injured, and number of deaths.^1^

**The Roy Search Method for India Human Stampede Events**

Roy and colleagues collected mass gathering data from news items reported in the archives of the three major India newspapers: The Times of India, The Hindu and The Indian Express. The search keywords were “stampede”, “mass gathering”, “mass-gathering events”, “mass-gathering incidents”, “crowd”, and “crowd management.” Data abstracted included location, triggers for the incident, number of deaths, and number of injuries from each incident.

Stampede events occurring in India between 2001 and 2010 found by the Ngai method were compared to those events found using the Roy method. Descriptive analysis was first performed for deaths and injuries including median and Pearson correlation coefficients. Bivariate analyses of number of deaths or injuries according to stampede characteristics were conducted using the nonparametric Wilcoxon rank test. Finally, a multivariate regression using a negative binomial model was used to account for overdispersion in the mortality data. Data were analyzed using Statistical Analysis Software (SAS) version 9.2 using PROC GENMOD (SAS Institute, Cary, NC).

## RESULTS

Between 2001 and 2010, a total of 40 human stampedes were identified by both methods. Of these, 34 human stampedes were identified using the Ngai method. Using a previously defined stampede scale: 2 events were class I, 21 events were class II, 8 events were class III, and 3 events were class IV (Table 1).^1^^,^ 10

**Table 1. India Human Stampede Events Characteristics 2001-2010.** 

**Class**	**Ngai Method**	**Roy Method**
I (No Deaths)	2	0
II (1-10 Deaths)	21	18
III (11-100 Deaths)	8	6
IV (101-1000 Deaths)	3	3
**Total No. Events**	**34**	**27**

The median deaths were 5.5 per event (range 0 to 267) and median injuries were 13.5 per event (range 0 to 300). Among the 34 events, 27 (79%) occurred during the daytime and 26 (76.5%) occurred indoor. With regard to the mechanism, 16 (47.1%) events were unidirectional, 26 (47.1%) events took place in the context of turbulent flow, and 4 (11.8%) were indeterminate. As for the type of event, 20 events (58.8%) were religious related, 6 (17.6%) were politically related, and 8 (23.5) were classified as “other.” (Table 2)

**Table 2. India Human Stampede Injuries and Fatalities.** 

	**Ngai Method**	**Roy Method**
Median Fatalities (IQR)	5.5 (2-17)	5 (2-39)
Median Injuries (IQR)	13.5 (5-31)	17 (10-45.5)
Pearson Correlation Coefficient	0.81 (p<0.001)	0.63 (P=0.003)

For the same time period, the Roy method identified a total of 27 events, including 9 unique events that were not identified by the Ngai method. Of these 9 events, three fell under Ngai’s exclusion criteria. Six additional events identified by the Roy’s method were all class I events, with a median of 4 deaths (range 1 to 8), and approximately 30 injuries (range 1 to 50, with 2 events yielding no injury data).

Bivariate and multivariate analyses were available for the Ngai database only due to the data extraction method. In bivariate analysis, unidirectional and turbulent mechanisms were found to be statistically different from indeterminate pedestrian traffic flow. However, there was no difference between unidirectional and turbulence mechanisms regarding deaths when indeterminate mechanisms were excluded from the analysis (Table 3).

**Table 3. Median Fatalities and Injuries by Characteristics of Events in India 2001-2010.** 

**Characteristics**		**Median Fatalities (IQR)**	**Median of Injuries (IQR)**
Day vs. Night			
	Day	6.0 (3-22)	12.0 (5-31)
	Night	2.0 (0-17)	20.0 (3-50)
Location			
	Outdoor	14.5 (5.5-27.5)	22.0 (5.5-56.5)
	Indoor	5.0 (2-9)	12.0 (5-30)
Type of Event			
	Others	2.5 (2-5)	9.0 (1-15)
	Religious	6.0 (4-23.5)	18.0 (8.5-49)
	Political	11.5 (1-22)	20.0 (1-37)
Mechanism			
	Turbulence	7.5 (4.5-22)*	11.5 (6.5-38)
	Unidirectional	5.0 (2-17)*	15.5 (10-37)
	Indeterminate	1.5 (0-2.5)	11.0 (1.5-19.5)
IQR = Interquatile range			
*P<0.05, nonparametric Wilcoxon rank sum test			

In multivariate analysis, religious and political events had higher relative number of deaths (Table 4).

**Table 4. Multivariate Analysis of Human Stampede Characteristics in India, 2001-2010.** *Multivariate negative binomial regression model excluding 4 events due to missing mechanism

**Characterstics**		**Relative No. Deaths (95% CI)**	**p-value**
Time of Day			
	Day	1.00	
	Night	1.88 (0.43-7.86)	0.408
Location			
	Outdoor	1.00	
	Indoor	1.92 (0.61-6.01)	0.268
Type of Event			
	Other	1.00	
	Religious	6.52 (1.73-24.66)	** 0.006**
	Political	277.09 (5.12-15001.96)	** 0.006**
Mechanism			
	Turbulence	1.00	
	Unidirectional	0.73 (0.23-2.32)	0.589

## DISCUSSION

Mortality and morbidity related to human stampedes are likely underestimated in the current literature.^1^^,^ 2 ^,^ 3 ^,^ 10 ^,^ 11 In this study, we explored and validated the need for improvement of reporting systems. The recent increased in the number of reported human stampedes is multifactorial. While improvement in public transportation and infrastructure may result in more crowding and larger events, the increasing number of reported events may likely reflect enhancements in global communication and eyewitnessed reports using smart phones and social media. Currently, surveillance of human stampedes remains in a retrospective fashion. With the advances in knowledge from other disasters like the Boston Marathon Explosions, crowd-sourced information may soon provide real time geocoding and confirmation of human stampedes events.^14^^,^ 15

In addition, improvement in our primary search method using LexisNexis® Academics also contributed to the increase in the number of events (Figure 1).

**Trend of Human Stampede Events and Reporting By Lexis Nexis® Academics, 1980-2012:**
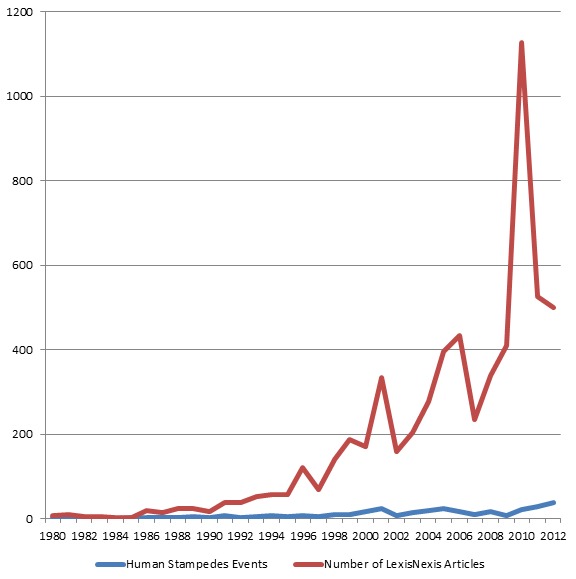

After comparing the two search methods, we concluded that the discrepancy was mostly attributable to the limitation of Lexis Nexis® Academics. Prior to 2010, Lexis Nexis® Academics did not include local newspaper, like the *Times of India*. As a result, the Ngai method underestimated stampede events in India by approximately 18%. No other comprehensive international database of human stampede events is publicly available or updated, to our knowledge.

## LIMITATIONS

There are several important limitations to this study. The exact number of deaths from human stampedes is presumed to have been accurately reported, but the number of injuries and participants remain unreliable, despite being a reasonable approximation.^1^^,^ 2 ^,^ 3 In addition, we applied the most conservative values for descriptive words such as “few” or “dozens”, a method we used in our previous report, thus likely resulting in underreporting.^1^

Finally, characteristics of each stampede event may be incomplete due to the limitation of internet and media sources, yet no other more accurate sources are currently available.^1^ While potential sources like hospital data, police reports, or funeral home records may improve the quality of the data, these require local resources and a standardized reporting network that is currently lacking.

## CONCLUSIONS

Many causes accounting for the global increase in human stampede events can only be elucidated through systematic epidemiological investigation. Focusing on a country with a high recurrence of human stampedes, we compared two independent methods of data abstraction in an effort to improve the existing database and to identify pertinent risk factors. As illustrated by the result of our study, an international standardized database to systematically record occurrence of human stampedes is needed to facilitate understanding of the epidemiology of human stampedes. While scientific explorations of human stampedes remain sparse, we should keep in mind the success of a country like Saudi Arabia in preventing crowd disasters during the annual Hajj and implement practices learned from Hajj for stampede prevention during other mass gathering events. Multidisciplinary collaboration including the systematic study of the physics behind crowd dynamics, structural engineering and infrastructure modifications, cultural factors, and progressive crowd control methods, combined with improved epidemiological understanding will be instrumental in effectively preventing future human stampede events.^2^^,^ 3 ^,^ 12 ^,^ 13
